# Characteristics of Shannon’s Information Entropy of Atomic States in Strongly Coupled Plasma

**DOI:** 10.3390/e22080881

**Published:** 2020-08-11

**Authors:** Myoung-Jae Lee, Young-Dae Jung

**Affiliations:** 1 Department of Physics, Hanyang University, Seoul 04763, Korea; mjlee@hanyang.ac.kr; 2 Research Institute for Natural Sciences, Hanyang University, Seoul 04763, Korea; 3 Department of Applied Physics, Hanyang University, Ansan, Kyunggi-Do 15588, Korea

**Keywords:** Shannon information entropy, strongly coupled plasma

## Abstract

The influence of shielding on the Shannon information entropy for atomic states in strong coupled plasma is investigated using the perturbation method and the Ritz variational method. The analytic expressions for the Shannon information entropies of the ground (1*s*) and the first excited states (2*p*) are derived as functions of the ion-sphere radius including the radial and angular parts. It is shown that the entropy change in the atomic state is found to be more significant in the excite state than in the ground state. It is also found that the influence of the localization on the entropy change is more significant for an ion with a higher charge number. The variation of the 1*s* and 2*p* Shannon information entropies are discussed.

## 1. Introduction

The investigation of the entanglement fidelity and Shannon information entropy has received considerable attention as it has been shown that the correlation effect plays an important role in understanding the quantum-measurements and information processing in physical systems [1,2]. It is also interesting to explore the influence of the coupling between the quantum state and the plasma density since the correlation effect will change the transfer of the quantum information in complex plasma systems. In strongly coupled plasma, the physical concept of Debye shielding based on the Debye–Hückel model cannot be applicable since the probability of finding plasma particles in a Debye sphere is almost negligible and the Debye number, i.e., the plasma parameter is smaller than the unity [3]. In a strongly coupled plasma system, the range of the interaction potential based on the ion-sphere model is strongly influenced by the constraint region defined by the ion-sphere radius since the potential would vanish beyond the size of the radius of the ion-sphere composed of a single ion and its surrounding negative-charge sphere [4]. Then, the atomic Shannon information entropy is expected to be determined by the localized shielding domain in strongly coupled plasma. However, the Shannon information entropy for atomic data in strongly coupled plasma has not yet been investigated. It is shown that the statistical entropy is related to the quantum-measurement of the correlation strength as a destructive property of many body systems [5,6]. In addition, the Shannon information entropy for atomic states is expected to provide the connection of the electron correlation with the statistical correlation [7]. Hence, in this research we investigate the localized correlation effects on the Shannon information entropy for atomic states in strongly coupled plasma using the ion-sphere model with an effective correlation distance. We then investigate the variation of the radial and angular parts of the atomic Shannon information entropies for the ground and the first excited states in strong coupled plasma as functions of the ion-sphere radius including electron correlations.

## 2. Theory and Calculations

In the ion-sphere model [3] of strongly coupled plasma, the interaction potential V(r) between an electron and an ion with nuclear charge *Ze* is given by

(1) $$V(r)\;=\;-\;\frac{Ze^{2}}{r}\;\left[1\;-\;\frac{r}{2R_{Z}}\;\left(3\;-\;\frac{r^{2}}{R_{Z}^{2}}\right)\right]\;\theta (R_{Z}\;-\;r)\;,$$

where $R_{Z}\;=\;{\left[3(Z\;-\;1)/4\pi n_{e}\right)}^{1/3}]$ is the radius of ion-sphere, $n_{e}$ is the number density of plasma electrons, and $\theta (R_{Z}\;-\;r)(\;=\;1\;\mathrm{for}\;R_{Z}\;\geq \;r;\;=\;0\;\mathrm{for}\;R_{Z}\;<\;r)$ is the Heaviside step function. The representation of the radial Schrödinger equation for the hydrogenic ion with the effective nuclear charge number $Z_{nl}(n_{e})(\;=\;Z\;-\;\delta_{nl})$ in strongly coupled plasma including the density effect can be justified as

(2) $$\frac{1}{r^{2}}\frac{d}{dr}\left[r^{2}\frac{dR_{nl}\left(r\right)}{dr}\right]\;-\;\frac{l\left(l+1\right)}{r^{2}}R_{nl}(r)\;+\;\frac{2m_{e}}{\hbar^{2}}\left[\frac{Z_{nl}(n_{e})e^{2}}{r}\;+\;E_{nl}\right]R_{nl}\left(r\right)\;=\;0,$$

here $\delta_{nl}$ is the shielding constant of the *nl*-shell by surrounding plasma electrons, $R_{nl}(r)$ is the radial wave function, $m_{e}$ is the electron mass, ℏ is the Planck constant, and $E_{nl}$ is the energy eigenvalue. Since the shielding constant is given by the effective Bohr radius $n^{2}a_{0}/Z_{nl}$, the expression of $\delta_{nl}$ can be obtained by $\delta_{nl}(Z)\;=\;\int_{0}^{n^{2}a_{0}/Z_{nl}}n_{e}d^{3}r$, where $a_{0}(\;=\;\hbar^{2}/m_{e}e^{2})$ is the Bohr radius of the hydrogen atom. The normalized variational 1*s* and 2*p* ansatzes can be written as $R_{1s}(r,\;\mu_{1s})\;=\;2\mu_{1s}^{-3/2}\;e^{-r/\mu_{1s}}$ and $R_{2p}(r,\;\mu_{2p})\;=\;(\mu_{2p}^{-\;5/2}/2\sqrt{6})\;r\;e^{-r/2\mu_{2p}}$, where the effective correlation distances $\mu_{1s}$ and $\mu_{2p}$ can be used as the 1*s* and 2*p* variation parameters. Using the perturbation method and the Ritz variational method [8], such as $\partial \langle E_{1s}(\mu_{1s})\rangle /\partial \mu_{1s}\;=\;0$, the energy expectation value $\langle E_{1s}(\mu_{1s})\rangle$ of the 1*s* state and $\delta_{1s}$ shielding constants are, respectively, found to be

(3) $$\langle E_{1s}(\mu_{1s})\rangle \;=\;\frac{\hbar^{2}}{2m\mu_{1s}^{2}}-\frac{(Z\;-\;\delta_{1s})e^{2}}{\mu_{1s}}\;,$$

(4) $$\delta_{1s}\;≅\;\frac{(Z\;-\;1){\left(a_{Z}/R_{Z}\right)}^{3}}{1\;-\;3(1\;-\;1/Z){\left(a_{Z}/R_{Z}\right)}^{3}}.$$

Hence, the 1*s* variation parameter, i.e., the effective correlation distance for the ground state, $\mu_{1s}$ is given by $\mu_{1s}(R_{Z})\;≅\;a_{Z}/[1\;-\;(1\;-\;1/Z){\left(a_{Z}/R_{Z}\right)}^{3}]$. For the 2*p* state, the energy expectation value $\langle E_{2p}(\mu_{2p})\rangle$ and $\delta_{2p}$ shielding constants are also obtained by the perturbation method and the Ritz variational method, i.e., $\partial \langle E_{2p}(\mu_{2p})\rangle /\partial \mu_{2p}\;=\;0$:

(5) $$\langle E_{2p}(\mu_{2p})\rangle \;=\;\frac{\hbar^{2}}{8m\mu_{2p}^{2}}-\frac{(Z\;-\;\delta_{2p})e^{2}}{4\mu_{2p}}\;,$$

(6) $$\delta_{2p}\;≅\;\frac{(Z\;-\;1){\left(4a_{Z}/R_{Z}\right)}^{3}}{1\;-\;3(1\;-\;1/Z){\left(4a_{Z}/R_{Z}\right)}^{3}}\;.$$

Then, the 2*p* variation parameter $\mu_{2p}$, regarded as the effective correlation distance for the 2*p* state, is found to be $\mu_{2p}(R_{Z})\;≅\;a_{Z}/[1\;-\;(1\;-\;1/Z){\left(4a_{Z}/R_{Z}\right)}^{3}]$. It is shown that the Shannon information entropy $S_{Shannon}$ [2,5,9] associated with the atomic density distribution $\rho (r,\;\Omega )[\;=\;{\left|R_{nl}(r)Y_{lm}(\Omega )\right|}^{2}]$ in a one-electron system is given by

(7) $$S_{Shannon}\;=\;-\;\int dr^{3}\;\rho (r,\;\Omega )\;\ln\rho (r,\;\Omega )\;=\;S(R_{nl})\;+\;S(Y_{lm}),$$

where $Y_{lm}(\Omega )$ represents the zonal harmonics, Ω represents the azimuthal and polar angles, $S(R_{nl})[\;=\;-\;\int dr\;r^{2}\;{\left|R_{nl}(r)\right|}^{2}\;\ln{\left|R_{nl}(r)\right|}^{2}]$ and $S(Y_{lm})[\;=\;-\;\int d\Omega \;{\left|Y_{lm}(\Omega )\right|}^{2}\;\ln{\left|Y_{lm}(\Omega )\right|}^{2}]$ are the radial and angular parts of the Shannon information entropy, respectively, and dΩ( = sinθ dθ dφ) is the differential solid angle in spherical coordinates. Hence, − lnρ(r, Ω) can be considered as the information contents since the entropy can be represented by the statistical averaged information content [10], such as $-\;S_{\rho }\;\propto \;\langle \ln\rho (r,\;\Omega )\rangle$.

For the 1*s* state of the hydrogenic ion in strongly coupled plasma, the angular part of the Shannon information entropy is $S(Y_{00})[\;=\;-\;\int d\Omega \;{\left|Y_{00}(\Omega )\right|}^{2}\;\ln{\left|Y_{00}(\Omega )\right|}^{2}]\;=\;\ln(4\pi )$ and the radial part of the Shannon information entropy $S(R_{10})$ for the 1*s* state is obtained as

(8) $$\begin{array}{lll} S(R_{10}) & \;=\; & -\;{\left(2\mu_{1s}^{-\;3/2}\right)}^{2}\;\int_{0}^{\infty }dr\;\ln{\left(2\mu_{1s}^{-\;3/2}e^{-\;r/\mu_{1s}}\right)}^{2}\;r^{2}\;e^{-\;2r/\mu_{1s}}\\ & \;=\; & \ln\left(\mu_{1s}^{3}/4\right)\;+\;3. \end{array}$$

The total Shannon information entropy for the 1*s* state is then found to be

(9) $$S_{1s}(\mu_{1s})\;=\;\ln\left[a_{Z}^{3}{\left[1\;-\;(1\;-\;1/Z){\left(a_{Z}/R_{Z}\right)}^{3}\right]}^{-\;3}\pi \right]\;+\;3.$$

Hence, the entropy change $\Delta S_{1s}[\;\equiv \;S_{1s}(\mu_{1s})\;-\;S_{1s}(a_{Z})]$ is then given by

(10) $$\Delta S_{1s}(R_{Z})\;=\;3\;\ln\left[{\left(1\;-\;(1\;-\;1/Z){\left(a_{Z}/R_{Z}\right)}^{3}\right)}^{-\;1}\right].$$

For the 2*p* state of the hydrogenic ion in strongly coupled plasma, the angular part of the Shannon information entropy $S(Y_{1m})\;=\;-\;\int d\Omega \;{\left|Y_{1m}(\Omega )\right|}^{2}\;\ln{\left|Y_{1m}(\Omega )\right|}^{2}$ should be evaluated for the $2p_{0}\;(m\;=\;0)$ and $2p_{\pm 1}\;(m\;=\;\pm \;1)$ substates so that $S(Y_{10})\;=\;\ln(4\pi /3)\;+2/3$ and $S(Y_{1,\pm 1})\;=\;\ln(2\pi /3)\;+\;5/3$. In addition, the radial part of the Shannon information entropy $S(R_{21})$ for the 2*p* state becomes

(11) $$\begin{array}{lll} S(R_{21}) & \;=\; & -\;{\left(\xi_{2p}^{-\;5/2}/2\sqrt{6}\right)}^{2}\;\int_{0}^{\infty }dr\;\ln{\left((\xi_{2p}^{-\;5/2}/2\sqrt{6}\;)\;r\;e^{-\;r/2\xi_{2p}}\right)}^{2}\;r^{4}\;e^{-\;r/\xi_{2p}}\\ & \;=\; & \ln\left(24\mu_{2p}^{3}\right)\;+\;2\gamma \;+\;\frac{5}{6}, \end{array}$$

where $\gamma \;=\;-\;\Gamma^{\prime }(1)(\;=\;0.5772157\;\ldots )$ is the Euler–Mascheroni constant [11]. The total Shannon information entropies for the $2p_{0}\;\left(m\;=\;0\right)$ and $2p_{\pm 1}\;\left(m\;=\;\pm 1\right)$ substates are now found to be, respectively,

(12) $$\begin{array}{lll} S_{2p_{0}}(\mu_{2p}) & \;=\; & \ln\left(32\pi \mu_{2p}^{3}\right)\;+\;2\gamma \;+\;9/6\\ & \;=\; & \ln\left[32\pi a_{Z}^{3}{\left(1\;-\;(1\;-\;1/Z){\left(4a_{Z}/R_{Z}\right)}^{3}\right)}^{-3}\right]\;+\;2\gamma \;+\;9/6, \end{array}$$

(13) $$\begin{array}{lll} S_{2p_{\pm \;1}}(\mu_{2p}) & \;=\; & \ln\left(16\pi \mu_{2p}^{3}\right)\;+\;2\gamma \;+\;15/6\\ & \;=\; & \ln\left[16\pi a_{Z}^{3}{\left(1\;-\;(1\;-\;1/Z){\left(4a_{Z}/R_{Z}\right)}^{3}\right)}^{-3}\right]\;+\;2\gamma \;+\;15/6, \end{array}$$

Hence, it is interesting to note that $S_{2p_{0}}(\mu_{2p})\;=\;S_{2p_{\pm 1}}(\mu_{2p})\;+\;\ln2\;-\;1$. Since the change of entropy $\Delta S_{2p}[\;\equiv \;S_{2p_{0}}(\mu_{2p})\;-\;S_{2p_{0}}(a_{Z})\;=\;S_{2p_{\pm 1}}(\mu_{2p})\;-\;S_{2p_{\pm 1}}(a_{Z})]$ is independent of the magnetic quantum number, it can be obtained as

(14) $$\Delta S_{2p}(R_{Z})\;=\;3\;\ln\left[{\left(1\;-\;(1\;-\;1/Z){\left(4a_{Z}/R_{Z}\right)}^{3}\right)}^{-1}\right].$$

Hence, it is expected that the simple analytic expressions of Equations (10), (12) and (13) provide the statistical averaged information contents for atomic states in strongly coupled plasma. Very recently, the thermodynamic properties of degenerate electron systems have been extensively explored, including the quantum phenomena such as the electron exchange-correlation, quantum diffraction, and quantum recoil effects [12,13,14,15,16,17,18]. Hence, the Shannon information entropy for atomic data investigated in degenerate quantum plasma will also be explored elsewhere.

## 3. Discussions

**Figure 1:**
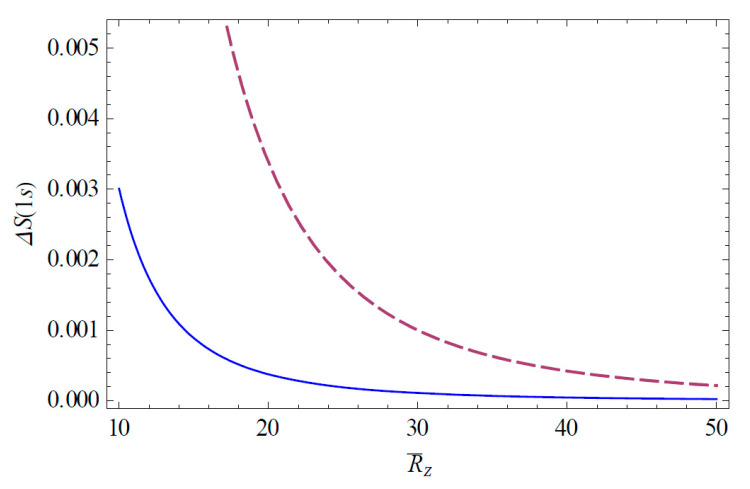
Entropy change $\Delta S_{1s}$ is plotted as a function of ${\bar{R}}_{Z}\;(\;=\;R_{Z}/a_{Z})$. The solid line shows the variation for Z = 2 and the dashed line shows the variation for Z = 10.

**Figure 2:**
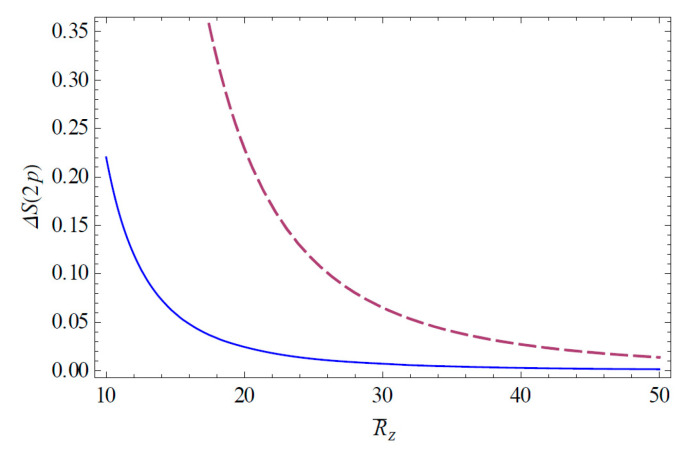
Entropy change $\Delta S_{2p}$ is plotted as a function of ${\bar{R}}_{Z}\;(\;=\;R_{Z}/a_{Z})$. The solid line shows the variation for Z = 2 and the dashed line shows the variation for Z = 10.

**Figure 3:**
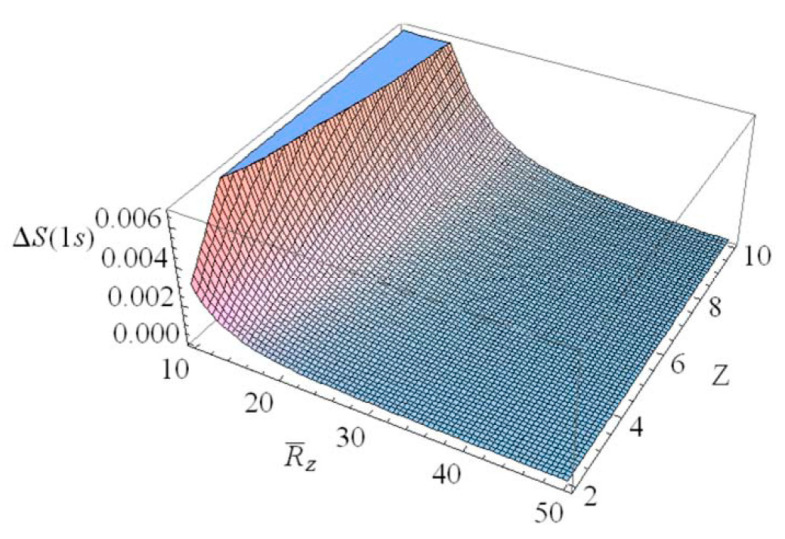
Three-dimensional plot of $\Delta S_{1s}$, drawn as a function of ${\bar{R}}_{Z}$ and Z.

**Figure 4:**
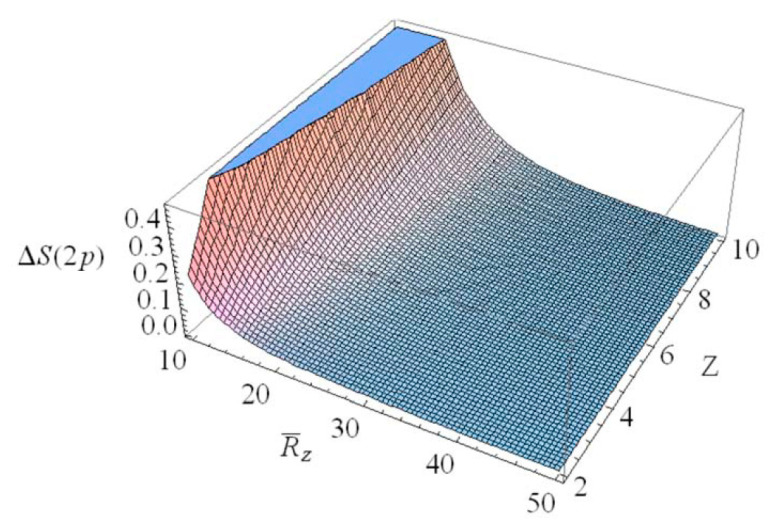
Three-dimensional plot of $\Delta S_{2p}$, drawn as a function of ${\bar{R}}_{Z}$ and Z.

**Figure 5:**
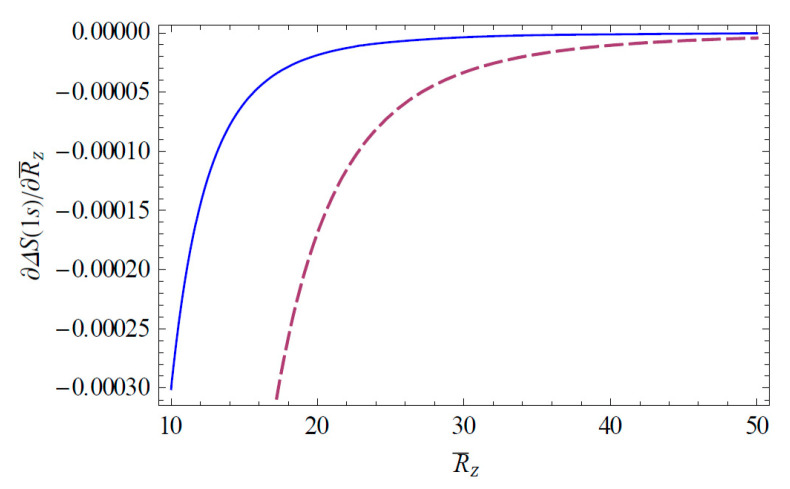
Derivative of $\Delta S_{1s}$ with respect to ${\bar{R}}_{Z}$, plotted as a function of ${\bar{R}}_{Z}$. The solid line shows the variation for Z = 2 and the dashed line shows the variation for Z = 10.

**Figure 6:**
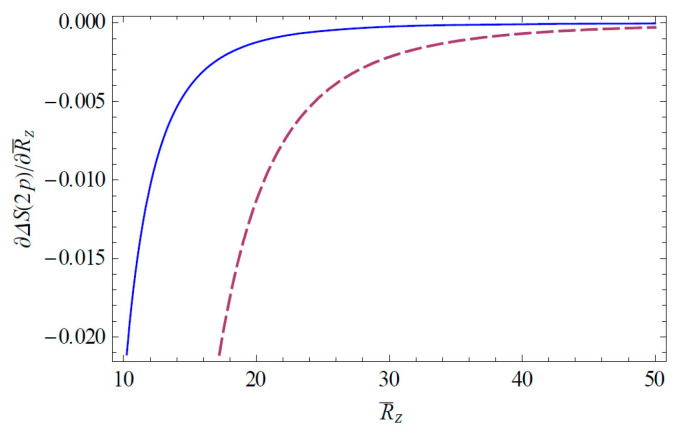
Derivative of $\Delta S_{2p}$ with respect to ${\bar{R}}_{Z}$, plotted as a function of ${\bar{R}}_{Z}$. The solid line shows the variation for Z = 2 and the dashed line shows the variation for Z = 10.

The Shannon information entropies of the hydrogen ion in strongly coupled plasma is expected to provide useful information on the transfer of the atomic data in a complex plasma environment. Figure 1 shows the change of the Shannon information entropy $\Delta S_{1s}$ for the 1*s* state in strongly coupled plasma as a function of ${\bar{R}}_{Z}\;(\;=\;R_{Z}/a_{Z})$. As we can see in Figure 1, the entropy change $\Delta S_{1s}$ decreases as ${\bar{R}}_{Z}$ increases. Hence, we have found that the strong localization of the ion-sphere radius strongly enhances the entropy change. It is also found that the entropy change $\Delta S_{1s}$ is enhanced with an increasing charge number. Figure 2 shows the change of the Shannon information entropy $\Delta S_{2p}$ for the 2*p* state as a function of ${\bar{R}}_{Z}$ in strongly coupled plasma. From Figure 1 and Figure 2, it is found that the entropy change in the atomic state is more significant in the excite state rather than the ground state due to the increase in the effective Bohr radius $\left(n^{2}a_{0}/Z_{nl}\right)$ in excited states. Figure 3 demonstrates the three-dimensional plot of the change of the Shannon information entropy $\Delta S_{1s}$ for the 1*s* state as a function of ${\bar{R}}_{Z}$ and Z. Figure 4 represents the three-dimensional plot of the change of the Shannon information entropy $\Delta S_{2p}$ as a functions of ${\bar{R}}_{Z}$ and Z in strongly coupled plasma. As it is seen in Figure 3 and Figure 4, the effect of localization on the entropy change is found to be more significant for an ion with a higher charge number. Figure 5 shows the radius-gradient of the entropy change $\partial \Delta S_{1s}/\partial {\bar{R}}_{Z}$ for the 1*s* state as a function of ${\bar{R}}_{Z}$ in strongly coupled plasma. Figure 6 indicates the radius-gradient of the entropy change $\partial \Delta S_{2p}/\partial {\bar{R}}_{Z}$ for the 2*p* state as a function of ${\bar{R}}_{Z}$. As we can see in these figures, the radius-gradient of the entropy change strongly decreases with an increase in the charge number in strongly coupled plasma. It is also shown that the domain of the negative change for the slop of the radius-gradient of the Shannon entropy for a given ion-sphere radius is strongly influence by the charge number of the ion in strongly coupled plasma.

## 4. Summary

In this research, we studied the influence of localized shielding on the Shannon information entropy of atomic states for strongly coupled plasma. We derived the analytic expressions of the Shannon information entropies for the ground and the first excited states as functions of the ion-sphere radius including the radial and angular parts by using the perturbation method and the Ritz variational method. The entropy change in the atomic state is found to be more significant in the excited state than in the ground state because of the increase in the effective Bohr radius in excited states. It has also been found that the influence of localization on entropy change is more significant for an ion with higher charge number. We conclude that shielding and localization play an important role in the Shannon information entropy in strongly coupled plasma. It is shown that the Shannon entropy [19] is the expected value of the information of a variable. Therefore, the Shannon atomic entropy of atomic states in plasma can provide information about atomic states as well as plasma parameters, such as the density and temperature. If we use plasma spectroscopy to investigate the collision and radiation processes in a plasma system, the Shannon atomic entropy would provide the mode detailed information on plasma parameters. In addition, the results of this study are expected to help researchers understand plasma through the diagnostics of strongly coupled plasma since the Shannon atomic entropy provides the complexity measure in the position space and the transmission of quantum information [1] about atomic collisions and radiation processes depends on the plasma conditions. These results should provide useful information on the transport of the atomic data and the physical characteristics of strongly coupled plasma.
